# Per- and polyfluoralkylated substances (PFAS) and other emerging contaminants in groundwater from central urban areas of São Paulo city, Brazil

**DOI:** 10.1007/s10661-026-15411-0

**Published:** 2026-05-05

**Authors:** Aluisio Soares, Reginaldo Antonio Bertolo, Mariana Amaral Dias, Luiz Guilherme Gomes Fregona, Jose Miguel Diaz Romero, Javier E. L. Villa, Cassiana C. Montagner

**Affiliations:** 1 EVA Way Environmental Projects, Rua Leôncio de Magalhães, 540, Jardim São Paulo, São Paulo, SP 02042-000 Brazil; 2 Groundwater Research Center (CEPAS), University of São Paulo (USP), 562, Rua Do Lago, São Paulo, SP 05508-080 Brazil; 3 Institute of Chemistry, University of Campinas, UNICAMP, Campinas, São Paulo, 13083-862 Brazil

**Keywords:** PFAS, Emerging contaminants, Groundwater, Contamination, Domestic effluent

## Abstract

The increasing demand for consumer products has led to the continuous introduction of novel substances into the environment. This contributes to contamination of environmental compartments and poses potential risks to human health. Many of these substances are classified as emerging contaminants and are detected at trace levels in various matrices, including groundwater. This study investigates the presence of emerging contaminants in shallow groundwater from a nonindustrial urban area potentially impacted by domestic wastewater, with an emphasis on per- and polyfluoroalkyl substances (PFAS). Twenty groundwater samples were collected in São Paulo city, Brazil, from older neighborhoods suspected of sewage collection system leakage, covering an entire hydrological cycle, that is, both dry and wet seasons. Samples were analyzed using liquid chromatography coupled with mass spectrometry (LC–MS/MS). PFAS were detected in concentrations ranging from 4.8 to 45.7 ng/L, with PFOS and PFOA being the most prevalent. Additionally, pesticides (1.1–736 ng/L), hormones (< LOQ–8.0 ng/L), pharmaceuticals (2.9–255 ng/L), and industrial compounds (14.1–314 ng/L) were also quantified in the samples. Although these concentrations are considered background levels associated with diffuse sources, the findings highlight the need for more comprehensive studies to assess the occurrence, sources, and potential health impacts of emerging contaminants in groundwater systems.

**Supplementary Information:**

The online version contains supplementary material available at 10.1007/s10661-026-15411-0.

## Introduction

With technological advancements driven by the growing demand for consumer goods, a wide variety of substances, comprising novel organic and inorganic compounds, are continually being developed to enhance specific properties and improve product appeal (Vélez et al., 2019). However, in many cases, these substances are introduced without adequate characterization of their interactions with environmental matrices or assessment of their potential toxicological effects on humans or ecosystems (Heberer, 2002; Ricart et al., 2010; Sui et al., 2015). Of particular concern are compounds classified as emerging contaminants (ECs). As studied over the last decades, these substances are defined as naturally occurring or anthropogenic chemicals that are not typically included in environmental monitoring programs but may be toxic, persistent, or capable of causing adverse or suspected effects in humans or animals, potentially altering biological functions (Sauvé & Desrosiers, 2014).

ECs comprise a broad spectrum of compounds from diverse applications, including their metabolites and transformation products. This group includes pharmaceuticals and personal care products, hormones, illicit drugs, per- and polyfluoroalkyl substances (PFAS), pesticides, disinfection by-products from drinking water and swimming pools, flame retardants, microplastics, veterinary medicines, industrial chemicals, and surfactants (Lapworth et al., 2012; Machado et al., 2016; Richardson & Manasfi, 2024; Stuart et al., 2012). Some ECs act as endocrine-disrupting chemicals (Kasonga et al., 2021) and others may impact the neurological system, potentially causing long-term effects (Montagner et al., 2017).

Environmental contamination by ECs may occur through direct or indirect pathways, such as the discharge of treated or untreated effluents (considering that conventional water treatment systems are generally ineffective at removing these substances), leaching from agricultural chemicals and landfills, by domestic daily uses, using flame-retardant foams, etc. (Lapworth et al., 2012; Stuart et al., 2012; Sui et al., 2015). As a result, ECs and their metabolites may reach the environment and accumulate in soil, surface water, groundwater, and even the atmosphere, posing risks to human health (Montagner et al., 2019; Alves et al., 2022; Zong et al., 2022; Murrel and Teehan 2021; Santos et al., 2025; Dias et al., 2025).

Among ECs, per- and polyfluoroalkyl substances (PFAS) are of particular concern. First developed in the 1940 s, PFAS have only faced regulatory scrutiny since the 1990 s, when studies began to demonstrate their persistence in the environment and potential human health impacts. These compounds are of global concern due to their environmental persistence, bioaccumulation potential, and associations with adverse health outcomes. PFAS have been detected in the human blood, liver, and lung tissue samples worldwide (Ahrens & Bundschuh, 2014; Kaiser et al., 2021; Li et al., 2025; Shearer et al., 2021; Souza et al., 2020; White et al., 2011).

PFAS are aliphatic compounds characterized by a carbon backbone in which hydrogen atoms are fully (per-) or partially (poly-) substituted with fluorine forming CnF_2n+1_ groups. Variations in terminal functional groups, such as carboxylates, sulfonates, sulfonamides, phosphates, and alcohols, confer a wide range of physicochemical properties (Buck et al., 2011; KEMI, 2015; Wang et al., 2020). Consequently, PFAS are extensively used in industrial and consumer applications, including heat-resistant food packaging, water- and stain-resistant fabrics, nonstick cookware, firefighting foams, cosmetics, adhesives, and pesticides (Giesy & Kannan, 2001; Jensen & Leffers, 2008; Rovira et al., 2019).

Among the thousands of PFAS compounds used in commerce, perfluorooctane sulfonic acid (PFOS) and perfluorooctanoic acid (PFOA) are the most widely studied due to their widespread use and carcinogenic potential (Jensen & Leffers, 2008; Wang et al., 2012; White et al., 2011). In 2005, PFOS was listed in Annex B of the Stockholm Convention as a persistent organic pollutant (POP). PFOA and its salts were included in Annex A in 2019, followed by perfluorohexane sulfonic acid (PFHxS) and related substances in 2022 (UNEP, 2023).

PFAS have been widely detected in multiple environmental compartments (Bach et al., 2017; Baabish et al., 2021; Cousins et al., 2022; Fiedler et al., 2022; Xu et al., 2025), and their strong carbon–fluorine bonds hinder degradation by conventional physical and chemical processes. As such, many PFAS plumes currently found in groundwater originate from releases that occurred decades ago, beginning with production by 3 M in 1949 (Ahmed et al., 2020; Xiao et al., 2015).

These substances can be transported long distances via atmospheric deposition, surface runoff, or groundwater flow (Ahrens & Bundschuh, 2014; Kurwadkar et al., 2022; Li et al., 2018). Studies have documented PFAS plumes extending between 0.5 and 5 km near known sources, such as firefighting foam training sites, nonstick product manufacturing plants, and treated wastewater recharge facilities (Moody et al., 2003; Nickerson et al., 2021; Bao et al. 2011; Cáñez et al., 2021).

In Brazil, most research on PFAS and other ECs has focused on surface water and public drinking water supplies (Carreri & Gehrke, 2024; Madeira et al., 2023; Starling et al., 2024). Groundwater studies are limited, particularly those assessing water supply systems (Stefano et al., 2022). Additionally, sulfluramid-based ant baits (EtFOSA) (Nascimento et al., 2018) are still permitted under Stockholm Convention exemptions.

This study aimed to evaluate the occurrence of PFAS and other emerging contaminants—including pharmaceuticals, hormones, and pesticides—in the shallow-groundwater aquifer layers from the city of São Paulo, potentially impacted by leaks from domestic sewage systems. Despite the extensive reliance on groundwater in São Paulo and its Metropolitan Region, with approximately 14,000 deep supply wells (30–300 m) producing about 10 m^3^/s (Bertolo et al., 2015), no studies have addressed emerging contaminants in groundwater; existing research has focused mainly on surface water and public supply systems (Coelho et al., 2020; Machado et al., 2016). Such investigations are necessary to assess the potential risk of contamination in deeper aquifers and to support the management of contaminated industrial sites (Pino et al., 2026), as the identification of these substances would provide essential baseline conditions for this specific urban environment.

## Experimental

### Study area and sampling

The city of São Paulo is in the hydrogeological context of the Alto Tietê Hydrographic Basin (BAT). This hydrological unit lies on sedimentary rocks of the São Paulo Basin, which are overlain and bordered by Precambrian rocks of the crystalline basement. The geotectonic unit that underlies the BAT is primarily composed of metasedimentary rocks, with minor occurrences of orthogneisses and magmatic rocks (Juliani, 1992). The sedimentary infill of the São Paulo Basin includes alluvial fan deposits (Resende Formation), lacustrine deposits (Tremembé Formation), meandering fluvial deposits (São Paulo Formation), braided fluvial deposits (Itaquaquecetuba Formation), and Quaternary surficial covers (Takiya, 1997).

The sedimentary units are grouped into three hydrogeological units: the Resende Aquifer, the São Paulo Aquifer, and the Quaternary Aquifer, collectively forming the Sedimentary Aquifer System (SAS) (Hirata & Ferreira, 2001; DAEE, 2009). This aquifer system ranges from unconfined to semiconfined, is highly heterogeneous, and exhibits primary porosity. The crystalline aquifer system (CAS) is subdivided into two units based on lithology (the granitic rock aquifer and the metamorphic rock aquifer) and is characterized as unconfined to semiconfined, heterogeneous, and anisotropic (Hirata & Ferreira, 2001). At the top of both aquifer units in the CAS, a thick weathered mantle is present, exhibiting distinct hydraulic behavior compared to the underlying solid rock. This weathered zone displays granular porosity, is generally unconfined, and is highly heterogeneous, with an approximate thickness of 50 m.

Groundwater samples were collected in a typical nonindustrial urban area of São Paulo city. According to the most recent census (IBGE, 2022), the city has a population of 11,451,999 inhabitants, a density of 7528.26 inhabitants/km^2^, and a total area of 1521.202 km^2^. Neighborhoods were selected based on their higher likelihood of contamination from domestic effluents, particularly due to leaks in the public sewage collection system. Therefore, sampling was prioritized in areas with higher population densities and older buildings, where the sewage infrastructure is outdated and more prone to leakage.

**Fig. 1:**
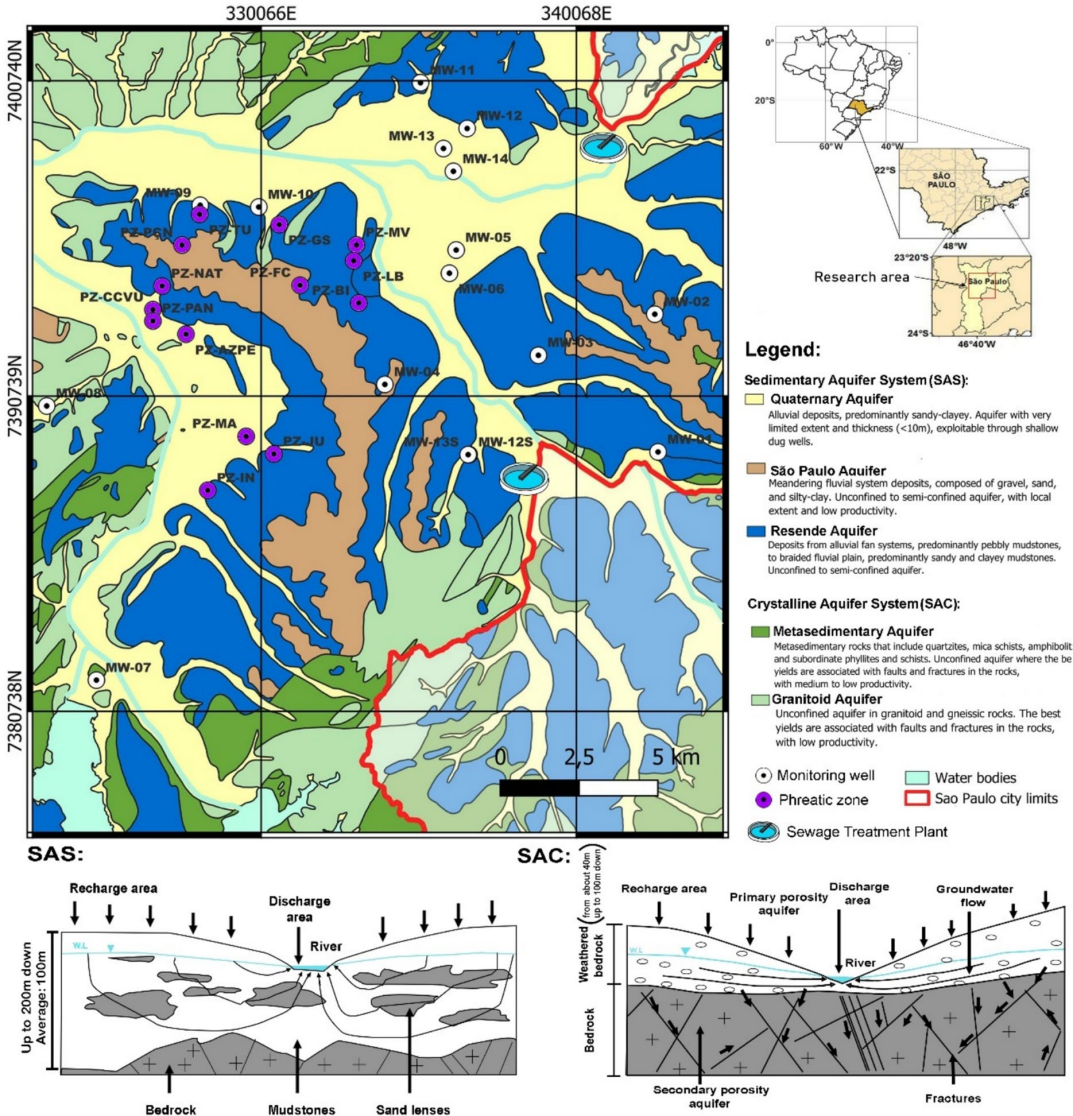
Map of the studied area in the city of São Paulo (São Paulo, Brazil) and the location of the groundwater sampling points, including monitoring wells (MW) and phreatic zone (PZ)

Groundwater samples were collected from preinstalled monitoring wells (MW) and from discharge points associated with phreatic zone drawdown (PZ), which is carried out for the construction of underground parking garages in buildings. These wells are relatively shallow (3–10 m) and are typically installed in the phreatic portion of the sedimentary aquifer system (Fig. 1). These depths contrast with those of deep supply wells, which have water inlets between 30 and 300 m deep. Shallow aquifer levels therefore act as potential sources of contamination for the deeper aquifer levels exploited by supply wells.

To identify potential contamination of shallow groundwater by domestic sewage, a preliminary screening campaign was conducted between October 2021 and June 2022. Thirty sampling points were selected for nitrogen species analysis (nitrate-N, nitrite-N, total Kjeldahl nitrogen (TKN), and ammoniacal nitrogen), including 16 from monitoring wells and 14 from the phreatic zone. Monitoring well (MW) samples were collected in peripheral neighborhoods, while phreatic zone (PZ) samples were collected in the central region of São Paulo city.

Considering nitrogen species as characteristic substances of domestic effluents, based on the results obtained in the screening campaign, the first sampling campaign for PFAS and other ECs was conducted during the dry season, which recorded 37.2 mm of rainfall (INMET, 2024). In August 2022, 10 samples were collected from monitoring wells and 10 from the phreatic zone. A second campaign took place during the wet season, with 234.8 mm of rainfall (INMET, 2024), in March 2023, including 10 MW samples and 10 PZ samples.

During the second sampling campaign conducted in 2023 (wet season), the sampling points MW-04, PF-MV, and PF-TU, previously collected during the 2022 campaign (dry season) with negligible PFAS and ECs, were replaced by PF-PÇN, PF-GS, and PF-CCVU. These replacement sites exhibited negligible concentrations of nitrate-N and ammoniacal-N during the screening campaign and were selected to evaluate potential correlations between the absence of sewage indicators and the absence of PFAS and other ECs in groundwater.

During all sampling campaigns, in situ physicochemical parameters such as potential of hydrogen (pH), oxidation–reduction potential (ORP), electrical conductivity (EC), dissolved oxygen (DO), and temperature were measured using a YSI Professional Plus multiparameter probe.

In accordance with Companhia Ambiental do Estado de São Paulo (CETESB, Environmental Company of the State of São Paulo) procedures, monitoring wells were purged prior to sampling. The purged volume was calculated based on screen diameter, total well depth, and groundwater-level depth, and three well volumes were removed. After groundwater-level stabilization, which varied according to the characteristics of each well, samples were collected using a dedicated sampler CETESB (2021). For monitoring wells, stagnant water was purged prior to sampling, and samples were collected using a dedicated sampler after the water level stabilized. Groundwater-level stabilization was assessed by measuring water levels before and after well purging. For phreatic zone samples, groundwater was collected directly from the discharge outlet located near the stormwater collection system using containers made of inert high-density polyethylene (HDPE).

Samples for nitrate-N and nitrite-N analysis were collected in 100-mL HDPE bottles without preservatives. Samples for ammoniacal nitrogen and TKN were collected in 500-mL HDPE bottles containing 3 mL of a 20% (v/v) sulfuric acid solution prepared with ultrapure water. For PFAS analysis, samples were collected in 250-mL HDPE bottles preloaded with 1 g of Trizma 1. Samples for other ECs were stored in 1-L amber bottles without preservatives. All collected samples were preserved in a refrigerator at 4 °C.

### Chemical analysis

Analyses were performed within 2 days for nitrate-N and nitrite-N, and within 28 days for ammoniacal nitrogen and TKN. Nitrate-N was analyzed using colorimetry in accordance with method SM24 4500 NO3 E:2022 (APHA et al., 2022). Nitrite-N was analyzed using the same method (SM24 4500-NO2 B:2022), but with NitriVer 3 reagent. Ammoniacal nitrogen was analyzed using an ammonia-selective electrode and the standard addition method (SM24 4500-NH3 E:2022). TKN analysis was conducted following SM24 4500-Norg C:2022 and SM24 4500-NH3 E:2022. While no sample preparation was required for nitrate-N, nitrite-N, or ammoniacal nitrogen, TKN analysis involved digestion and subsequent distillation.

PFAS analyses were performed within 14 days following USEPA Method 537.1 (USEPA, 2020). Samples (250 mL) were extracted using InertSep MA-2 SPE cartridges after conditioning with methanol and phosphate buffer (pH 3). Following sample loading at a controlled flow rate, cartridges were dried and analytes were eluted with ammonium hydroxide in methanol. The eluate was concentrated under nitrogen, reconstituted in methanol/water (80:20, v/v), spiked with internal standards, filtered, and analyzed by UPLC-MS/MS using a BEH C18 column.

The other ECs were quantified using a method previously developed and validated by solid-phase extraction with Oasis HLB 500 mg (Waters) cartridges and liquid chromatography coupled with mass spectrometry (LC–MS/MS) (Acayaba et al., 2021; Dias et al., 2025; Montagner et al., 2014; Santos et al., 2022). Briefly, samples were filtered through a glass fiber filter (Sartorius 47 mm diameter, grade 13,400) and loaded onto HLB cartridges previously conditioned with methanol and ultrapure water. Then, methanol and acetonitrile were used to elute the samples, and the extract was dried completely under a gentle nitrogen flow. Samples were resuspended in a mixture of methanol:ultrapure water (30:70, % v/v) and filtered through a hydrophobic polytetrafluoroethylene syringe filter (0.22 µm, 13 mm diameter). The final extracts were analyzed on an LC–MS/MS (Agilent LC 1200, MS 6410B).

The supplementary material provides Table S2, presenting all analyzed substances, along with detailed descriptions of the analytical methods and the limit of quantification (LOQ) for all target contaminants at ng/L levels.

### Quality assurance and quality control (QA/QC)

PFAS sampling was conducted using HDPE bottles and PFAS-free equipment to minimize the risk of cross-contamination. For the other ECs, amber glass bottles were previously decontaminated with 1% aqueous Extran solution and rinsed with ultrapure water, ethanol, and acetone. Afterward, the bottles were placed in a muffle furnace for 4 h at 400 °C and capped with aluminum foil. For PFAS and other ECs, laboratory blanks were analyzed alongside the samples to detect potential cross-contamination. During LC–MS/MS analysis, solvent solutions in vials were used between defined injections to prevent carryover.

### Multivariate data analysis

Exploratory data analysis using principal component analysis (PCA) and self-organizing maps (SOM) was performed after excluding parameters with uniform and informative values across all samples (e.g., the same LOD) and auto-scaled to prevent variance differences caused by unit differences. Both algorithms were implemented in Python, specifically using the scikit-learn and MiniSom libraries. Visualization of the results (e.g., SOM projection, score, and loadings plots) was accomplished using the matplotlib library. Additionally, 95% confidence ellipses were applied to the PCA score plots to provide a statistical context for the group separations observed.

## Results and discussion

### Physicochemical parameters

The results of the physicochemical parameter for the three sampling campaigns (screening, wet, and dry) are presented in Table S3 of the supplementary material. The ORP (oxidation–reduction potential) values measured in situ were corrected for E_H_ (redox potential) according to Jardim (2014). Considering the screening campaign, pH values ranged from 4.14 (MW-09) to 7.20 (PZ-MV), electrical conductivity ranged from 88.7 (PZ-GS) to 930 µS/cm (MW-12), dissolved oxygen from 1.00 (MW-14) to 9.00 mg/L (PZ-NAT), redox potential (E_H_) from 77.4 (MW-02) to 539 mV (PZ-FC), and temperature ranged from 18.2 (PZ-NAT) to 28.1 °C (MW-06). These results show that most of the groundwater samples have pH values within a range considered neutral, i.e., between 5.5 and 8.5, with conductivity values demonstrating the presence of dissolved ions, as well as oxidizing environments, with significant dissolved oxygen values and positive E_H_ values.

According to Jardim (2014), positive E_H_ values indicate oxidizing conditions and are consistent with the nitrate-N concentrations obtained. According to Freeze and Cherry (1979) and Hudak (2000), nitrate-N is the form of nitrogen that prevails in oxidizing groundwater, due to its stability and high mobility, which allows it to move according to advective flow and not be adsorbed by soil particles.

### Nitrogen species

The results about nitrogen species are presented in Tables S4(a) and S4(b) in the supplementary material. There are several possible anthropogenic sources of these contaminants in groundwater, including agricultural activities, solid waste disposal, sanitation systems, and domestic effluents from, for example, leaking sewage systems. Studies have reported that the main contaminant of the nitrogen species in groundwater from domestic sewage is nitrate-N (Montanheiro & Kiang, 2016; Zhang et al., 2015; Grimmeisen et al., 2017; Vystavna et al., 2017). The presence of nitrate in groundwater contaminated with domestic effluents also indicates an advanced degree of organic matter degradation, as this substance reflects the final stages of oxidation of raw effluent (Macêdo, 2003).

The concentrations obtained specifically for nitrate-N ranged from 0.271 (PZ-GS) to 32.8 mg/L (MW-11). Considering the reference value of 10 mg/L, recommended by GM/MS Ordinance No. 888, of May 4, 2021 (Brazil, 2021), which sets potability standards in Brazil, concentrations above this value were found in 23% of the samples (MW-04, MW-06, MW-09, MW-11, PZ-AZPE, PZ-NAT, and PZ-MA). However, for all the samples analyzed, the concentrations of nitrate-N were above the LOQ (0.1 mg/L), indicating the potential alteration of groundwater quality, mainly due to leaks from domestic sewage. In a study conducted by Varnier et al. (2010) in the city of Marília/SP, high concentrations of nitrate-N were also found, especially in the older neighborhoods of the city (central zone), which have higher urban density. This suggests a link between the urbanization process and the nitrate-N load present in the aquifer, likely to originate from old cesspools and leaks in the sewage collection networks.

Although there are no guideline values for TKN, all the concentrations obtained were also above the LOQ (0.05 mg/L), with the MW-05 (19.8 mg/L) and MW-12 (13.4 mg/L) samples standing out, indicating the presence of organic nitrogen. For nitrite-N, all the results were below the USEPA (2024) guideline value of 2.0 mg/L, indicating that the samples are oxidized, since the nitrogenous forms present in sewage are converted into ammonia, nitrite, and nitrate during oxidation. It should be noted that in Brazil, there are no guideline values for nitrite-N. However, the presence of this contaminant in the samples also indicates the presence of domestic sewage. The levels of ammoniacal nitrogen in domestic sewage are due to the hydrolysis of organic nitrogen (primarily urea), thereby corroborating the results obtained for TKN.

The correlation between the sum of the nitrogen species and pH is shown in Figure S1 in the supplementary material. The acidity caused by nitrification processes leads to a decrease in pH values (Tchobanoglous et al., 2003). In other words, the negative correlation of pH with the total nitrogen sum indicates that most groundwater samples underwent redox processes resulting in nitrification, revealing the presence of domestic effluents, and confirming the possibility of leaks from the sewage collection network, which is in direct contact with the aquifer’s water samples.

### Spatial distribution and concentration profiles of PFAS

Based on the results obtained for nitrogen species, which indicated the possibility of contamination by domestic sewage, considering the concentrations primarily of nitrate (as N), 20 samples were selected for PFAS analysis during the season of lowest rainfall. In addition, to verify the direct correlation between the presence of PFAS in groundwater contaminated with domestic sewage, three samples were collected during the season of highest rainfall, whose concentrations of the nitrogen species were not significant in the screening sampling event.

**Fig. 2:**
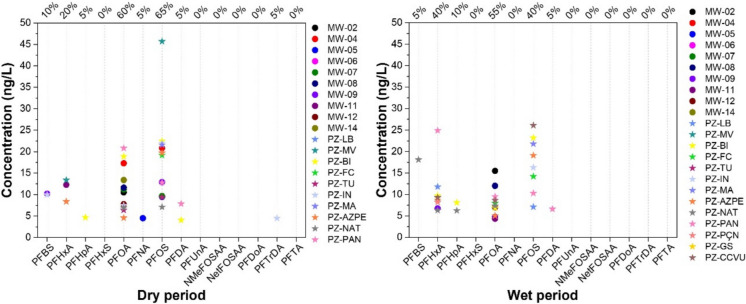
PFAS concentration (ng/L) and detection frequency for samples collected during the dry and wet seasons (*n* = 23)

The concentrations of PFAS obtained for both seasons of higher (wet) and lower (dry) rainfall are described in Fig. 2 and Tables S5(a) and S5(b) in the supplementary material. The results indicate that 8 PFAS were quantified above their respective limits of quantification in the groundwater samples collected during both seasons: PFOS, PFOA, PFHxA, PFBS, PFNA, PFHpA, PFDA, and PFTrDA.

Considering PFAS guideline values established by the USEPA in the regional screening level (RSL) resident tapwater table (target risk = 1 × 10^–6^, target hazard quotient = 1) (USEPA, 2024), all concentrations obtained above the LOQ for PFOA are also above the guideline limit established based on risk assessment (2.7 × 10^–2^ ng/L). It should be noted that, in this case, the LOQ is also above this limit, as the chemical analyses were conducted before the RSL was updated in May 2024. For PFOS, concentrations above the RSL were also obtained for the following samples: MW-04, PZ-MV, PZ-BI, PZ-MA, and PZ-CCVU. It is noteworthy that, in 83% of the samples (*n* = 40), at least one PFAS was detected above its respective LOQ, indicating the presence of these substances throughout the groundwater of the aquifer. For the sake of comparison, since Denmark was the first European country to ban the use of PFAS and uses 98% of its water from underground reservoirs for human consumption (Danish Ministry of the Environment, 2004), a comparison was made with its guideline values. It is evident that the results obtained are all below these values, but it should be noted that they are less restrictive than the values recommended by USEPA.

The predominant PFASs during the dry season were PFOS and PFOA, whose concentrations represented detection frequencies of 65% and 60% of the samples, respectively. For this season, PFOS concentrations varied between 7.10 and 45.7 ng/L while PFOA varied between 4.59 and 18.9 ng/L. For the wet season, in addition to PFOS (40%) and PFOA (55%), significant concentrations were also obtained for PFHxA in 40% of the samples, whose concentrations varied for PFOS between 7.11 and 26.1 ng/L, for PFOA between 4.36 and 15.5 ng/L, and for PFHxA between 6.30 and 24.9 ng/L.

Wei et al. (2018), in a study conducted in a nonindustrial region of Jiangsu Province, China, also reported a high frequency of detection of PFOA and PFOS. Similarly, the PFAS detection rates obtained are consistent with the observations from a study carried out in eastern China (Chen et al., 2016) and in Hanoi and Ho Chi Minh City, where PFOA and PFOS were the primary compounds detected in groundwater (Duong et al., 2015). PFOA and PFOS are also the most frequently detected substances in domestic wastewater treatment stations (Barisci & Suri, 2021; Li et al., 2023), indicating that these substances are present in many materials commonly used in households, such as fabric waterproofing agents, carpets and upholstery, nonstick cookware, cleaning products, and even personal care items like shampoos and cosmetics.

**Fig. 3:**
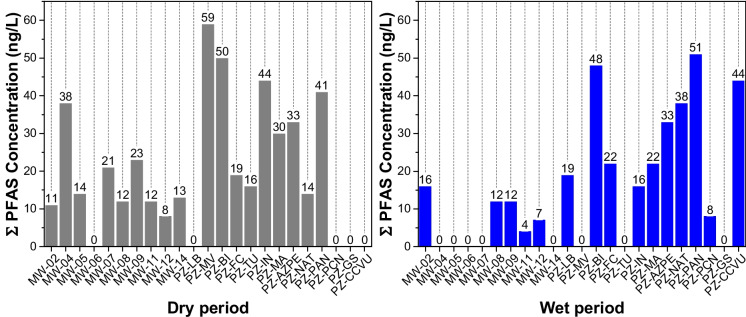
Sum of PFAS concentrations (ng/L) at each sampling site in monitoring wells (MW) and phreatic zone (PZ) during the dry (gray bars) and wet (blue bars) seasons between October 2021 and June 2022. The zero values above the bars indicate that no PFAS were detected at the sampling point

Figure 3 shows the sum of PFAS data for each sampling point considering the wet and dry seasons. It is evident that the highest concentrations were obtained for the samples collected in the phreatic zone located in neighborhoods considered the oldest in the city of São Paulo, reflecting the greater possibility of leakage from the older domestic effluent collection network in these neighborhoods. Bräunig et al. (2017) and Wang et al. (2015) indicated that PFAS in groundwater come mainly from domestic sewage (such as food packaging, cosmetics, pharmaceuticals and human/animal urine/feces, etc.), atmospheric deposition, and pollution from nearby industrial parks. Specifically in Brazil, regarding household applications, the use of sulfluramid for controlling termites, cockroaches, and household ants has exceeded, for several years, the specific exceptions established by the Stockholm Convention, which may also contribute to the presence of PFAS in domestic sewage.

Considering the results obtained for the dry and wet seasons, the sum of PFAS concentrations at several sampling sites (MW-08, MW-12, PF-BI, PZ-FC, and PZ-AZPE) did not exhibit significant seasonal differences. In contrast, marked seasonal variability was observed at other sites, with higher concentrations during the dry season at MW-05, MW-07, MW-09, MW-11, MW-14, PZ-IN, and PZ-MA, and higher concentrations during the wet season at MW-02, PZ-NAT, and PZ-PAN. Overall, the majority of sampling points exhibited higher PFAS concentrations during the dry season. Similar seasonal behavior was reported by Xu et al. (2025), who observed higher sum of PFAS concentrations in groundwater during dry periods. Van Horne et al. (2019) also reported higher PFAS concentrations during the dry season. Both studies identify factors that may account for these differences, such as source type, proximity to the source, and the hydrological characteristics of the aquifers.

Thus, during the sampling campaign conducted in the dry season, reduced recharge of the unconfined aquifer was observed, resulting in lower dilution and, consequently, higher sum of PFAS concentrations. Considering that the sampling sites exhibiting elevated PFAS concentrations during the dry season also showed higher nitrate-N concentrations, the results indicate the influence of domestic sewage inputs as a potential source of contamination.

### Other emerging contaminants

**Fig. 4:**
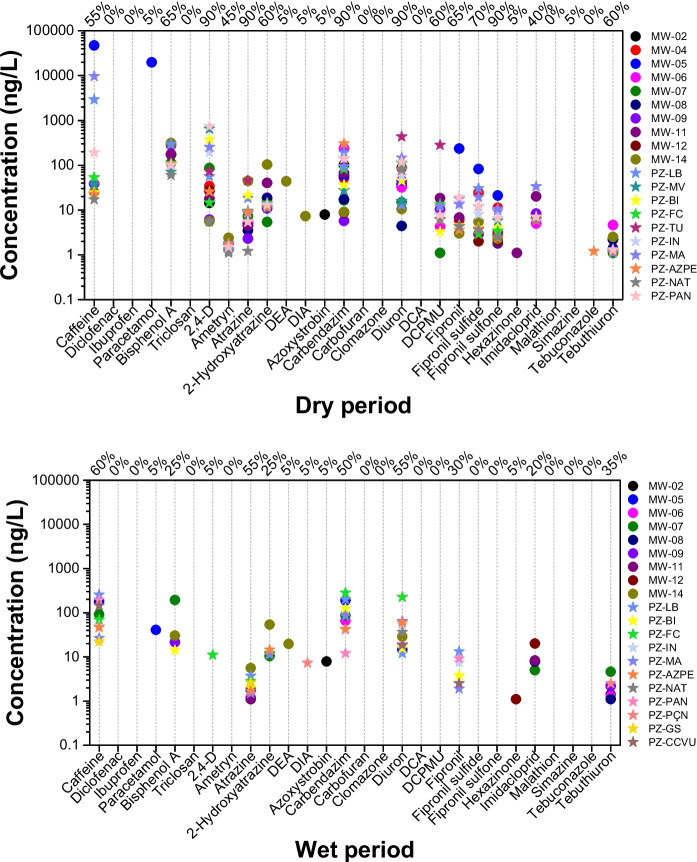
Results for the other emerging contaminants (ECs) from the samples collected during the dry and wet seasons

The concentrations determined for the other ECs in both seasons are described in Fig. 4 and Tables S5(a) and S5(b) in the supplementary material. For pharmaceuticals compounds, caffeine had a maximum concentration of 47 ng/L (MW-05), as well as paracetamol, with a point concentration of 20 ng/L (MW-05). Hormones were quantified in only one sample at concentration of 8.0 ng/L (MW-02), while for industrials, the maximum concentration of bisphenol A was 313 ng/L (MW-14). In the other hand, pesticides were quantified in many samples, with 736 ng/L of 2.4-D (PZ-PAN) and 434 ng/L of Diuron (PZ-TU) standing out.

For the sampling carried out during the wet season, caffeine showed a maximum concentration of 255 ng/L (PZ-LB). In contrast, for the hormones, no substance exceeded the LOQ. For industrials, Bisphenol A showed a maximum concentration of 194 ng/L (MW-07), while for pesticides, several substances also showed concentrations above the LoQ, with carbendazim standing out at 283 ng/L (PZ-FC) and diuron at 226 ng/L (PZ-FC).

It should also be noted that during the dry season, 90% of the samples showed concentrations of 2,4-D, atrazine, carbendazim, diuron, and fipronil sulfone, whereas during the wet season, the frequency of detection was lower, with 55% of the samples containing diuron and atrazine. Specifically, caffeine was detected in 55% and 60% of the samples for the dry and wet seasons, demonstrating that this substance is constantly present in domestic sewage and, consequently, in groundwater. Based on these results, similarly to what was observed for the summed PFAS concentrations, it is evident that higher concentrations, as well as a greater number of detected emerging contaminants (ECs), were observed during the dry season.

Based on Ordinance GM/MS No. 888 of May 4, 2021 (Brazil, 2021), which establishes the reference values for drinking water quality in Brazil, it was found that the concentrations of certain compounds analyzed in this study, including 2,4-D, atrazine, carbendazim, and diuron, did not exceed their respective regulatory limits.

**Fig. 5:**
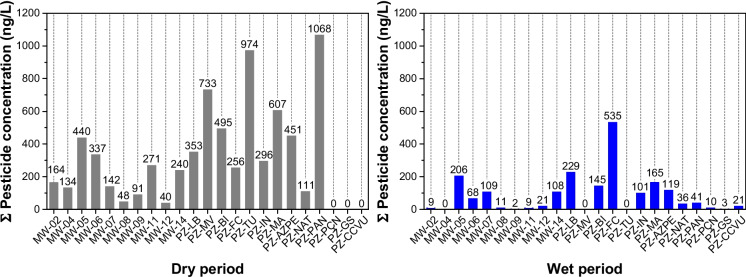
Sum of pesticide concentrations (ng/L) at each sampling site in monitoring wells (MW) and phreatic zone (PZ) during the dry (gray bars) and wet (blue bars) seasons between October 2021 and June 2022. The zero values above the bars indicate that no pesticide was detected at the sampling point

Considering only the sum of the pesticides presented in Fig. 5, including the sampling campaigns carried out during both the dry and wet seasons, it is observed that they are uniformly distributed across all the samples analyzed, as was also observed for PFAS.

All sampling points are in an area of the city of São Paulo characterized by high population density, consisting of old and well-established neighborhoods. This setting results in substantial generation of domestic wastewater and a high likelihood of leakages in the sewer network, as evidenced by the results obtained, particularly elevated nitrate-N concentrations. Consequently, increased contaminants, such as pesticides and PFAS, to the groundwater system are also expected.

The results indicate that groundwater contamination is predominantly diffuse, as no significant differences were observed between sampling points located near wastewater treatment plants, which would represent a possible point-source of contamination (Fig. 1). This behavior contrasts with the findings reported by Xu et al. (2025), who observed pronounced spatial differences in PFAS concentrations in groundwater samples collected near a fluorochemical plant in China, characterizing a point-source contamination scenario.

Similarly to the results observed for PFAS, higher concentrations of other emerging contaminants, particularly pesticides, were also detected during the dry season. This pattern supports the interpretation that reduced aquifer recharge during dry periods enhances the influence of domestic sewage inputs, resulting in increased contaminant concentrations due to lower dilution.

### Exploratory data analysis

PCA on MW and PZ datasets effectively differentiates groups mainly via the first two components: PC1 accounts for 23.56% of the total variance, PC2 for 12.92%. The scores plot shows PC1 is key for distinguishing MW and PZ (Fig. S2a). Loadings reveal variables influencing separation: for MW, PC1 is driven by PFAS (PFHxA, PFOA, and PFHpA), TKN, conductivity, and temperature. These factors suggest that the MW group is characterized by a significant presence of PFAS compounds, alongside other key environmental parameters associated with contamination and water quality, such as ammoniacal-N, which indicates the influence of domestic sewage, as well as electrical conductivity, which reflects the presence of dissolved ions in the water, also derived from domestic sewage.

The PZ group is linked to physicochemical parameters like EH, ORP, and DO on PC1, indicating more dynamic redox conditions that influence contaminant mobility and persistence in the phreatic zone. This highlights environmental and contamination differences between groups. The MW group relates to PFAS, emphasizing the importance of these contaminants in monitoring wells, while the PZ group’s association with redox parameters suggests redox processes primarily affect phreatic wells. Sampling conditions also differ: MW samples, collected with a bailer, are stable, whereas PZ samples, pumped directly from sidewalks, reflect more sensitive redox conditions.

PCA on MW and PZ datasets shows clear differentiation between these groundwater sources. PC1 and PC2 account for 49.54% of variance, with PC1 at 30.37% and PC2 at 19.17%. The score plot reveals PC1 effectively separates MW and PZ, indicating it as the main axis of differentiation (Figure S3a). The separation reflects distinct environmental and contamination profiles. The loading plot for PC1 shows parameters influencing this separation, with PFOA, PFHpA, PFHxA, and TKN most impactful for MW. These PFAS pollutants suggest MW is affected by contamination from areas with PFAS use.

TKN measures both organic nitrogen and ammonia, indicating nitrogenous compounds’ role in MW’s water chemistry. Its presence signifies nutrient pollution, leading to eutrophication and poorer water quality. The PZ group is separated on PC2, mainly affected by N-nitrite and PFDA. N-nitrite, linked to domestic sewage, signals nitrogen pollution in PZ wells, implying contamination from sewage leaks. PFDA, a persistent bioaccumulative PFAS, emphasizes environmental impact on the PF group.

PCA analysis of MW and PF datasets reveals that PC2 and PC3, accounting for 34.09% of variance (16.59% and 13.23%, respectively), effectively differentiate MW and PZ groups by capturing key variations. The score plot shows that PC2 distinctly separates MW from PZ, with PC3 further reinforcing this distinction (Figure S4a). The PC2 loading plot shows that the MW group is mainly influenced by TKN and N-nitrate (Figure S4b), compounds linked to domestic sewage and impacting groundwater quality. Their influence suggests proximity to nitrogen pollution sources. The PZ group is associated with herbicides and pesticides like atrazine, carbendazim, diuron, 2,4-D, as well as N-nitrite, indicating nitrogen cycling driven by microbial activity in groundwater. The PC3 loading plot shows MW is mainly affected by tebuthiuron and bisphenol A, indicating industrial and domestic sewage influence. The PZ group remains linked to 2,4-D and N-nitrite, signs of herbicide and fertilizer use, emphasizing their vulnerability to domestic sewage.

To evaluate the relationship between PFAS and pesticide concentrations, an exploratory analysis using self-organizing maps (SOM) was conducted. SOM does not assume linear relationships among variables, and it has proven efficient at extracting comprehensive and key information from the data. The topological and quantization errors were 0.025 and 0.27, respectively, indicating that the SOM provided a suitable representation of the data. As shown in Fig. S5, the MW and PZ samples were partially separated into the upper and lower regions of the projection map (consisting of 24 hexagonal neurons), respectively, underscoring the ability to uncover patterns in the data. A complementary assessment of the features in Figure S6 displays the distribution of the features (i.e., pesticides and PFAS) for the samples formerly projected in the SOM map. This highlights the excellent interpretability of SOM and indicates a strong correlation between pesticide and PFAS concentrations, with particular emphasis on PFOS and PFOA.

A study conducted by Donley et al. (2024) in the USA and Canada identified the presence of PFAS in several commonly used pesticides, which may be indicative of the results obtained in this study regarding the presence of these two classes of contaminants in groundwater samples contaminated by domestic sewage.

## Conclusions

This study evaluated the occurrence of PFAS and a broad suite of emerging contaminants (pharmaceuticals, hormones, pesticides, and industrial compounds) in shallow urban groundwater of São Paulo, with the aim of assessing potential inputs from leaking domestic sewage and implications for deeper supply aquifers and also establishing baseline concentrations in a strongly urbanized area.

PFAS and other emerging contaminants were widely detected across the sampled shallow aquifers. PFAS occurred in 83% of samples, with PFOS and PFOA the most frequent (maximum concentrations of 45.7 ng/L and 18.9 ng/L, respectively). Other PFAS (PFHxA, PFBS, PFNA, PFHpA, PFDA, and PFTrDA) and multiple pharmaceuticals, pesticides, hormones and industrial chemicals were also quantified at trace to low ng/L levels. Spatial patterns showed higher PFAS loads in older central neighborhoods, and concentrations were generally higher in the dry season, consistent with reduced dilution. Positive associations between elevated nitrate/TKN and PFAS support domestic sewage as a major source for many detected compounds.

The study’s main contributions are threefold: (1) it provides the first systematic evidence of PFAS and diverse emerging contaminants in São Paulo’s shallow urban groundwater, addressing a documented knowledge gap; (2) it demonstrates the effectiveness of combining simple sewage indicators (nitrogen species) with targeted contaminant screening to prioritize sampling and infer sources; and (3) it highlights the vulnerability of shallow aquifers to diffuse urban effluents and the potential for contaminant migration toward deeper municipal supply wells.

Future work should prioritize (1) expanded monitoring across a larger, stratified network of shallow and deep wells to map plume extent and vertical migration; (2) improved analytical sensitivity for key PFAS to meet current guideline thresholds; (3) process studies on attenuation and transport in the weathering mantle and fractured bedrock; (4) exposure and risk assessment linking occurrence data to water-use patterns.

In summary, the findings indicate pervasive low-level contamination of São Paulo’s shallow groundwater by PFAS and other emerging contaminants, underscoring the need for expanded surveillance, enhanced analytical capacity, and targeted management to protect groundwater resources and public health.

## Supplementary Information

Below is the link to the electronic supplementary material.

ESM 1 (DOCX 871 KB)

## Data Availability

No datasets were generated or analysed during the current study.
