# Supplementary material for: Per- and polyfluoralkylated substances (PFAS) and other emerging contaminants in groundwater from central urban areas of São Paulo city, Brazil
Source: Environ Monit Assess. 2026 May 5;198(5):548. doi: 10.1007/s10661-026-15411-0 (PMC13139244; doi:10.1007/s10661-026-15411-0)
Supplement: Supplementary file 1 — (DOCX 871 KB) [file 10661_2026_15411_MOESM1_ESM.docx]

**Journal:** [**Environmental Monitoring and Assessment**](https://link.springer.com/journal/10661)

**Per- and poly-fluoralkylated substances (PFAS) and other emerging contaminants in groundwater from** **central urban areas of São Paulo city, Brazil**

**Aluisio Soares^1,2^, Reginaldo Antonio Bertolo^2^, Mariana Amaral Dias^3^, Luiz Guilherme Gomes Fregona^2^, Jose Miguel Diaz Romero^3^, Javier E. L. Villa^3^, Cassiana C. Montagner^3*^**

^1^ EVA Way Environmental Projects, Rua Leôncio de Magalhães, 540, Jardim São Paulo, São Paulo, SP, 02042-000

^2^ Groundwater Research Center (CEPAS), University of São Paulo (USP), Rua do Lago, 562, São Paulo, SP, 05508-080, Brazil.

^3^ Institute of Chemistry, University of Campinas, UNICAMP, Campinas, São Paulo, 13083-862, Brazil

1. Soares

[aluisio.soares@evaway.com.br](mailto:aluisio.soares@evaway.com.br)

R. A. Bertolo

[bertolo@usp.br](mailto:bertolo@usp.br)

M. A. Dias

mariad@unicamp.br

L. G. G. Fregona

luiz.fregona@usp.br

J. M. D. Romero

j272422@dac.unicamp.br

J. E. L. Villa

[jelv@unicamp.br](javascript:;)

*C. C. Montagner (corresponding author)

ccmonta@unicamp.br

https://orcid.org/0000-0002-6475-5969

**Study Area and Sampling**

**Table S1:** Sampling site locations

**Chemical Analysis**

**Analytical methods**

**Nitrogen Species**

Nitrate concentrations were determined using a colorimetric method in accordance with Standard Methods 4500-NO₃⁻ E:2022. In this procedure, 10 mL of the sample were transferred to a 25 mm diameter cylindrical glass cuvette, followed by the addition of a NitraVer 5 reagent sachet. After a 5-minute reaction period, absorbance was measured at 534 nm using a DR 2800 spectrophotometer (Hach).

Nitrite analysis adhered to Standard Methods 4500-NO₂⁻ B:2022, employing the same colorimetric approach with the substitution of NitraVer 5 by NitriVer 3 reagent. The reaction time was extended to 20 minutes, and absorbance readings were taken at 400 nm.

Ammonia nitrogen (NH₃-N) concentrations were quantified using an ammonia-selective electrode method with standard addition, following Standard Methods 4500-NH₃ E:2022. A 100 mL aliquot of homogenized sample was combined with 1 mL of EDTA solution (prepared by dissolving 400 g NaOH and 45.2 g EDTA in ultrapure water to a final volume of 1 L). The mixture was continuously stirred, and measurements were taken using an Orion 9512 ammonia-selective electrode coupled with an Orion 3 Star millivolt meter (Thermo Scientific). An initial millivolt reading was recorded, followed by the addition of 10 mL of an ammonia standard solution with a concentration ranging from 1 to 1000 mg/L, depending on the initial reading. After stabilization, a second millivolt reading was obtained. The ammonia concentration was calculated based on the change in millivolt readings and the known concentration of the added standard.

Total Kjeldahl Nitrogen (TKN) was measured following Standard Methods 4500-Norg C:2022 and 4500-NH₃ E:2022. A 25 mL aliquot of the sample was digested with 10 mL of digestion solution (comprising 134 g K₂SO₄, 7.3 g CuSO₄, and 134 mL H₂SO₄, diluted to 1 L with ultrapure water). The mixture was homogenized and heated on a digestion block at 375–385°C for 30 minutes post vapor release. After cooling, 10 mL of distillation solution (prepared by dissolving 500 g NaOH and 25 g Na₂S₂O₃ in ultrapure water to a final volume of 1 L) were added to the digestate. Steam distillation was performed using ultrapure water in a nitrogen distillation apparatus (TE-0364, Tecnal). The distillate was collected in 0.04 N H₂SO₄ solution up to a volume of 50 mL. Subsequently, the distillation apparatus was removed from direct contact with the solution, and distillation continued for an additional 2 minutes. The TKN concentration was determined using the same ammonia-selective electrode method described above, with the only modification being the use of a 20 mL aliquot of the collected 0.04 N H₂SO₄ distillate.

**PFAS**

After activating the InertSep MA-2 cartridge (GL Sciences, Japan) by conditioning with 10 mL of methanol followed by 10 mL of 0.1 M sodium phosphate buffer, the cartridge—containing approximately 3 mL of buffer solution—was topped up with deionized water and acidified with acetic acid to reach pH 3. The vacuum was adjusted to allow a dropwise flow rate. Then, 250 mL of each sample were passed through the extraction cartridges, and the inner walls of the sample bottle were rinsed with 10 mL of ammonium acetate solution (1 g L⁻¹). Subsequently, 1 mL of methanol was added to the sample bottle. The cartridges were then left to dry for at least 60 minutes. Elution was carried out using 4 mL of ammonium hydroxide in methanol, initially added into the sample bottle, followed by another 4 mL of the same solution, carefully rinsing the bottle walls with this solvent.

Next, 8 mL of the eluate were transferred to a concentrator tube. The sample bottle walls were additionally rinsed with 2 mL of the same ammonium hydroxide solution. The methanol was evaporated to dryness in a 60 °C water bath under a nitrogen stream. The extract was then reconstituted in 1.0 mL of methanol/water (80:20, v/v), followed by the addition of 34 µL of an internal standard solution. The solution was filtered through a 0.45 µm membrane filter prior to injection into an ultra-performance liquid chromatography system tandem mass spectrometry (UPLC-MS/MS, Xevo TQD, Waters, USA). Analytes were separated on a BEH C18 column and subsequently identified using a mass spectrometer.

**Other Emerging Contaminants (ECs)**

Immediately after the collection, the samples were filtered under vacuum using a glass fiber membrane (Sartorius grade 13,400 – Gottingen, Germany). The samples were extracted by solid-phase extraction (SPE) using HLB Oasis cartridges 500 (Waters Corporation – Milford, USA), according to a method previously developed and validated (Montagner et al., 2014, Acayaba et al., 2020, Santos et al., 2022, Dias et al., 2025). Briefly, SPE cartridges were conditioned with methanol and ultrapure water. Then, 500 mL of water samples were loaded, followed by elution with methanol and acetonitrile. The solvent was dried in N2 flow until dryness, and then the extract was reconstituted using 500 µL of a mixture of water:methanol 70:30 (v/v). The extract was filtered through a polytetrafluoroethylene (PTFE) hydrophobic syringe filter with a porosity of 0.22 µm and analyzed by liquid chromatography coupled to mass spectrometry in tandem (LC-MS/MS). All the chromatographic conditions were optimized, and the validation parameters can be found in previous publications.

**Results**

**Physicochemical parameters**

**Nitrogen Species**

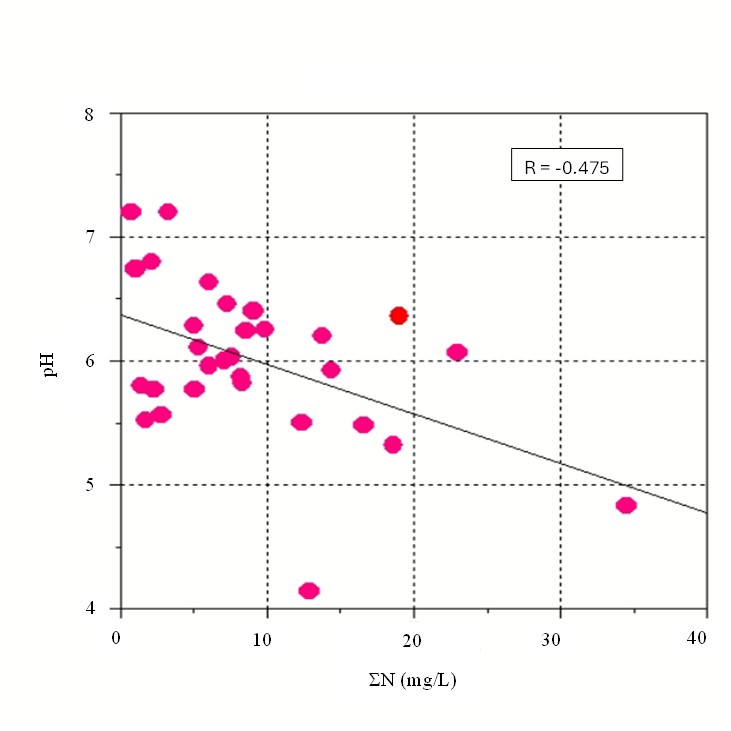


**Fig S1.** Scatter plot of the pH and sum of nitrogen species from the screening campaign

**Spatial distribution and concentration profiles of PFAS**

**Other Emerging Contaminants**

**Exploratory data analysis**


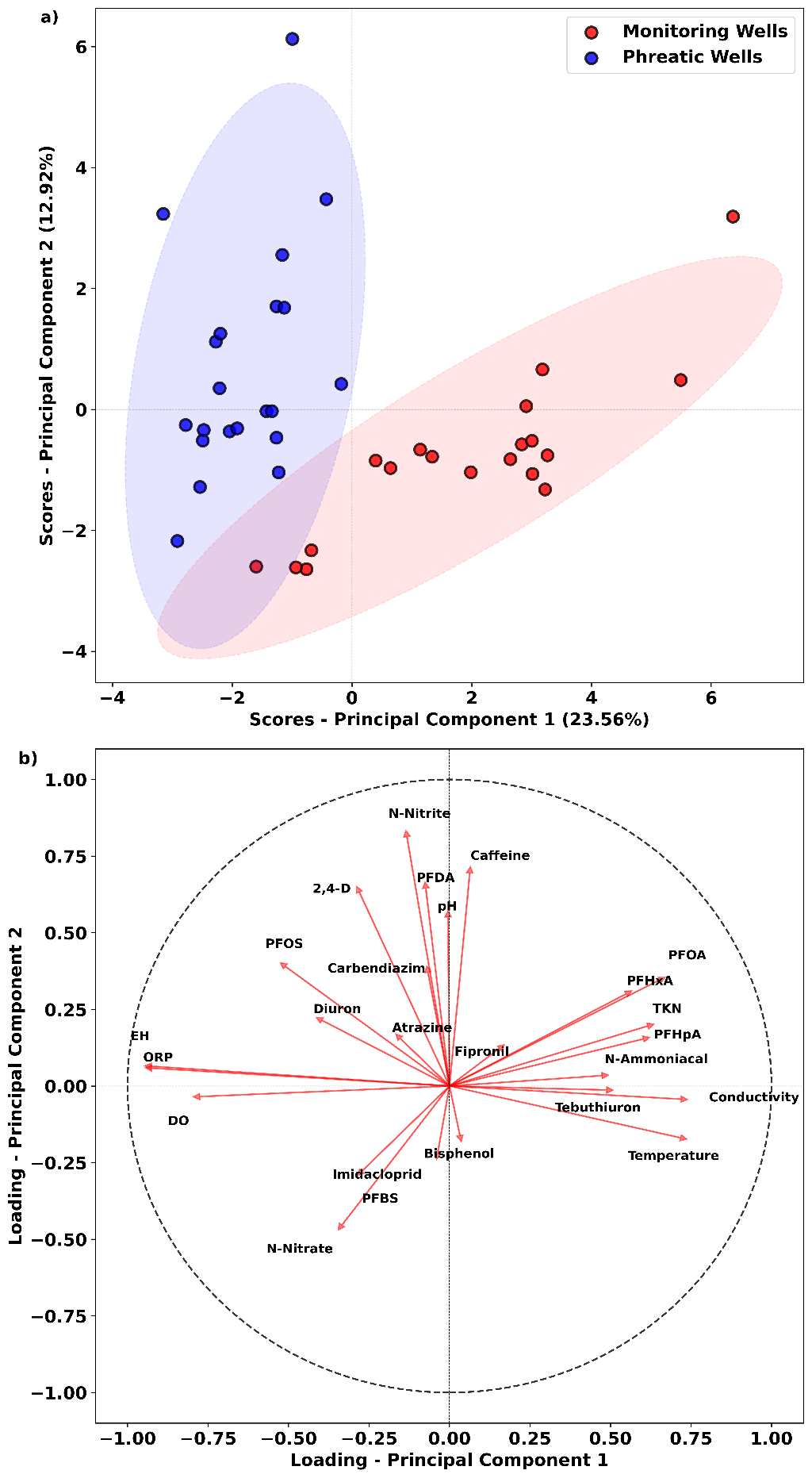


**Fig. S2** PCA results of samples collected from monitoring wells (MW) and phreatic wells (PZ). The scores plot (a) visualizes the distribution of samples along the first two principal components, the loadings plot (b) shows the contribution of each parameter.


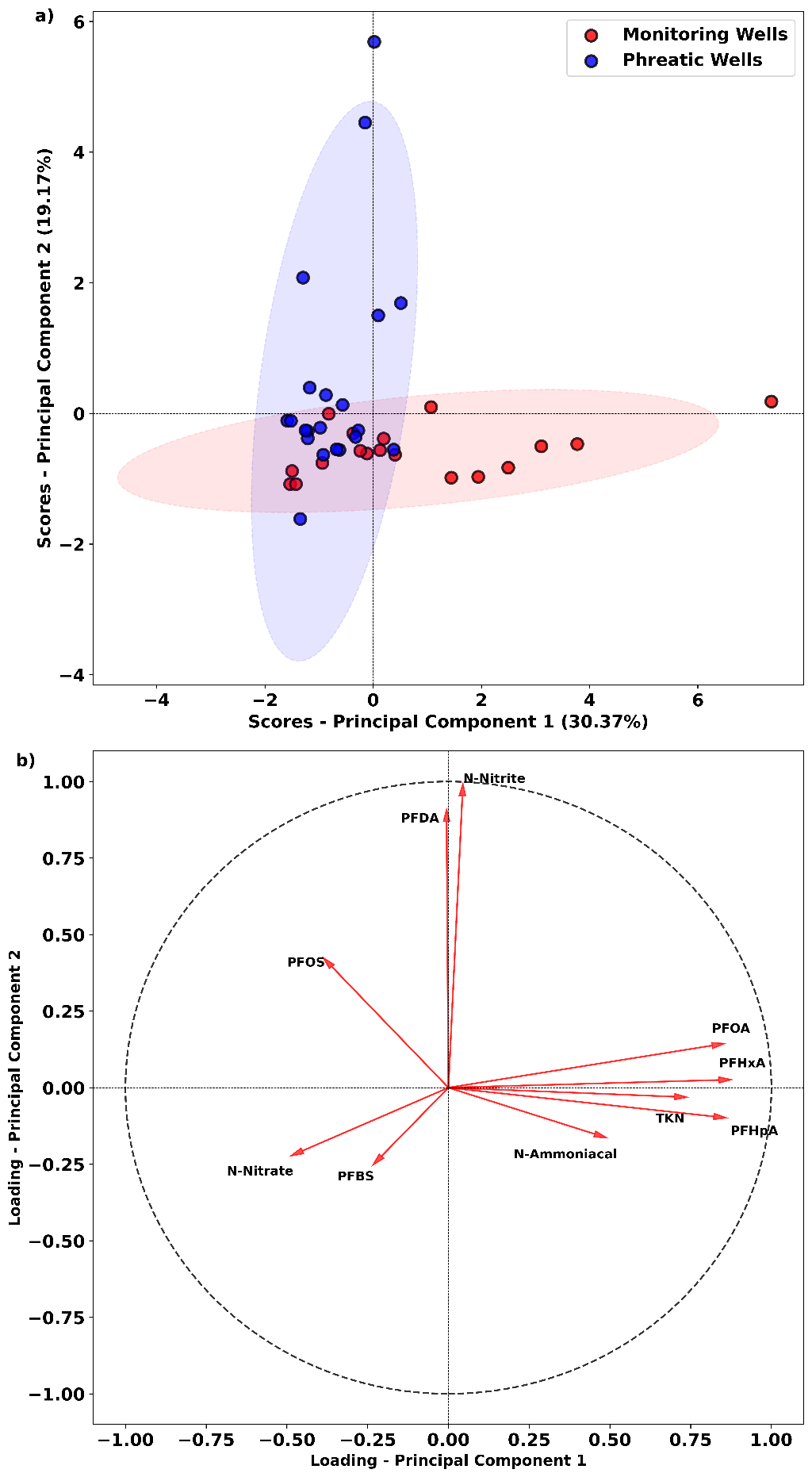


**Fig. S3** PCA results of samples collected from monitoring wells (MW) and phreatic wells (PZ). The scores plot (a) visualizes the distribution of samples along the first two principal components, the loadings plot (b) shows the contribution of each parameter

**
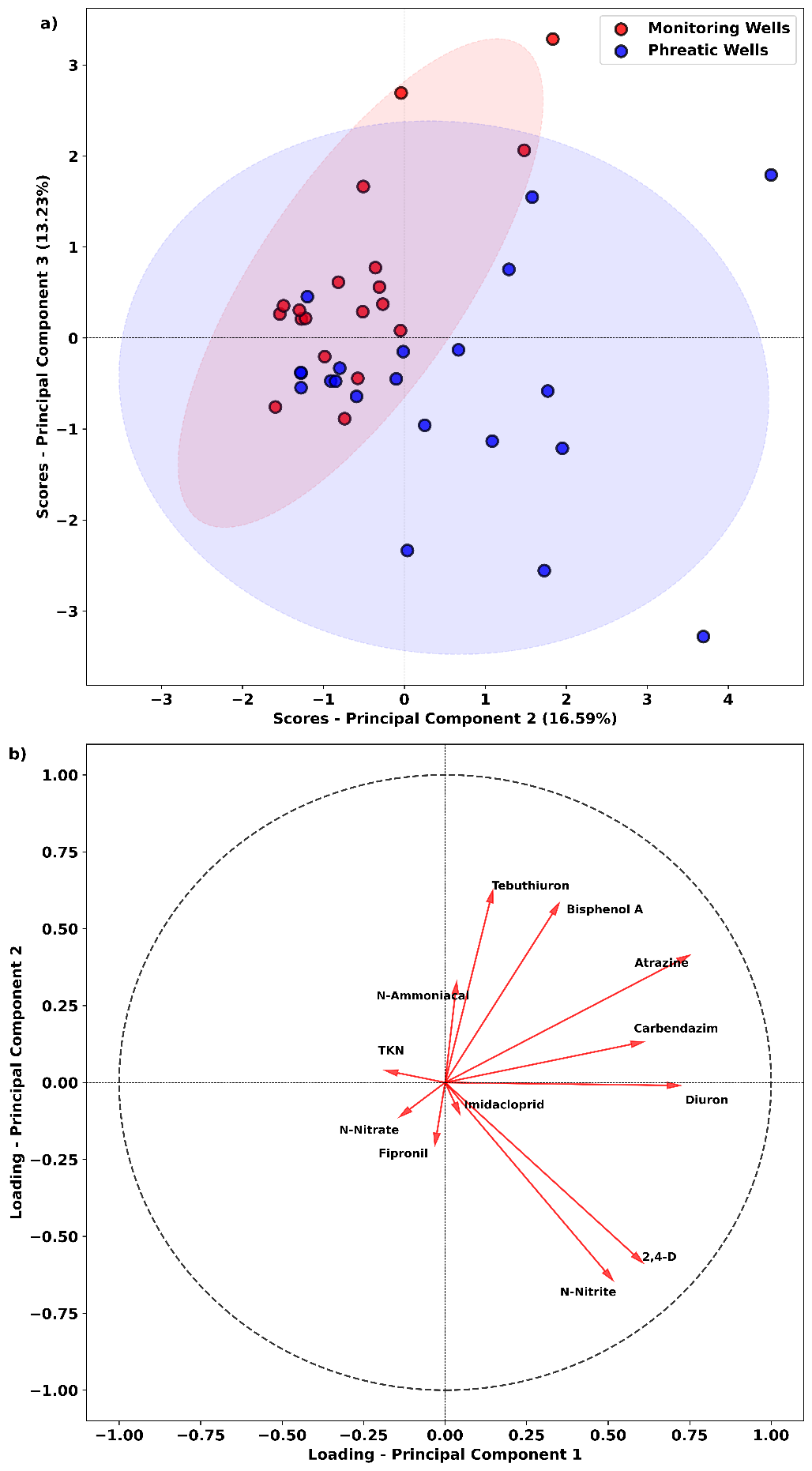
**

**Fig. S4** PCA results of samples collected from monitoring wells (MW) and phreatic wells (PZ). The scores plot (a) illustrates the distribution of samples along Principal Components 2 (PC2) and 3 (PC3). (b) The loadings plot shows the contribution of each parameter to these principal components.


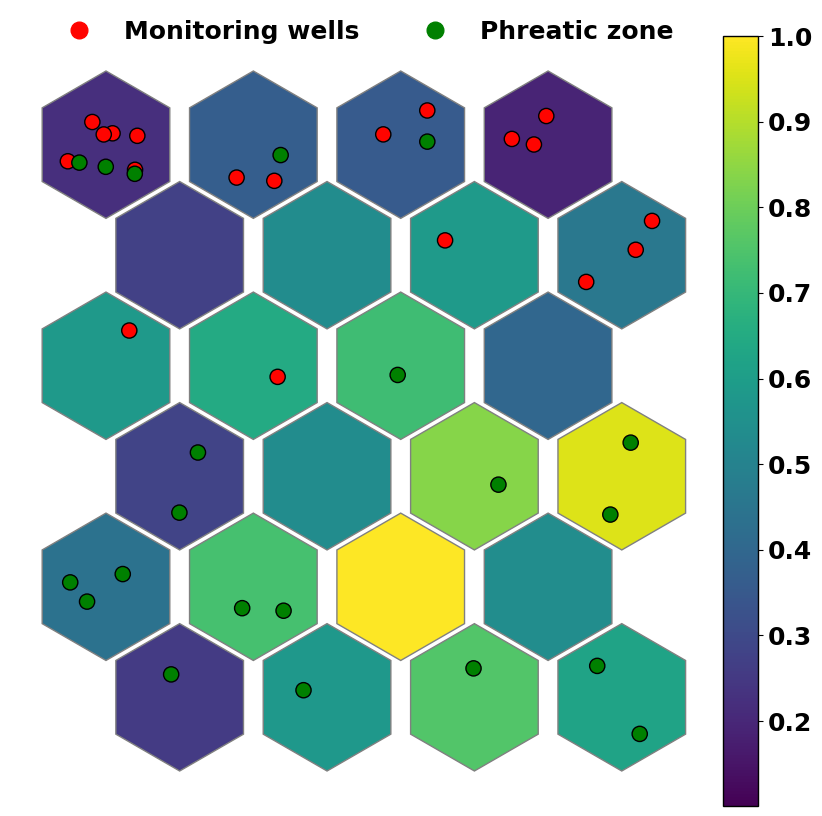


**Fig S5** Projection of the samples onto the bidimensional normalized SOM subspace.


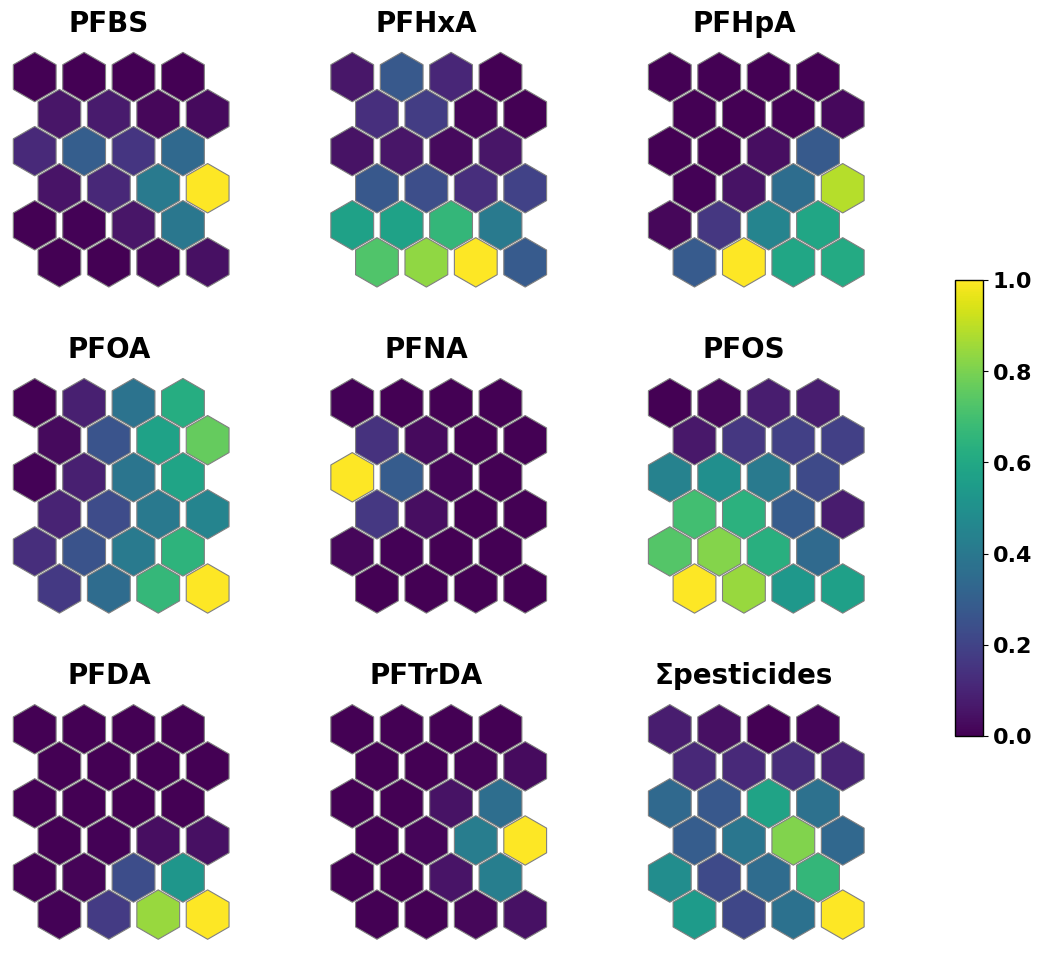


**Fig S6.** Component planes for the PFAS and pesticides for the assessment of feature importance.
